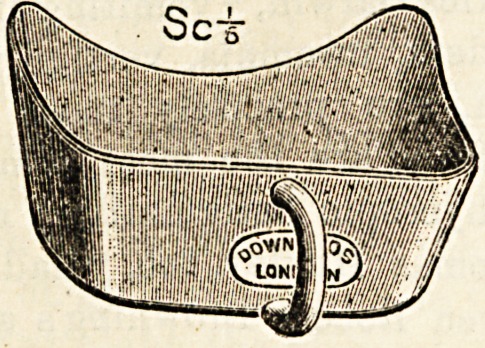# New Appliances and Things Medical

**Published:** 1903-05-23

**Authors:** 


### NEW APPLIANCES AND THINGS MEDICAL.

### NEW KARNOID PREPARATIONS.

(Karnoid, Limited, 6 and 7 Stonecutter Street, Ludgate Circus, London, E.C.)

We have received from the above firm two new preparations which, owing to the addition of certain nutritive principles obtained by the combination of small proportions of milk, wheat, and malt, must be regarded as distinct improvements on the earlier preparations of this firm which we have examined. The new Karnoid meat preparations which we have tested are (1) beef, (2) chicken powder; each of these is a dry powder soluble to the extent of 50 per cent. in cold water, and consisting of a meat basis of about 60 per cent. When dissolved in water this powder makes a very delicious cup of soup, highly palatable, nutritious, and stimulating. Since these Karnoid meat preparations contain a high percentage of proteid matter they are strongly to be commended for invalids and others who require their bodily strength restored by the building up of sound physiological tissues. Proteid foods are practically the only articles of diet which can accomplish this object, but the majority of such foods are unsuitable for invalids with impaired powers of digestion. These Karnoid preparations are suitable for such extreme cases of gastric insufficiency as ulcer of the stomach.

### NURSE MANCHESTER'S OPHTHALMIC DRESSING TRAY.

(Down Brothers, Limited, St. Thomas's Street, Borough, London, S.E.)

The accompanying illustration represents a dressing tray which has been specially designed for use in the irrigation of the eyes. It is so constructed that it fits accurately to the side of the face, and hence can be used for patients in bed without fear of the lotion finding its way among the bed-clothes. This tray is cleanly, light, and handy to use, and Nurse Manchester is to be congratulated on her ingenuity in satisfying such an obvious want in ophthalmic surgery.

## Figures and Tables

**Figure f1:**